# A myostatin inhibitory antibody combined with insulin, partially rescues the musculoskeletal phenotype of female insulin-deficient diabetic mice

**DOI:** 10.3389/fendo.2025.1558740

**Published:** 2025-06-12

**Authors:** R. Clay Bunn, Reuben Adatorwovor, Philip D. Ray, Alexander R. Keeble, Christopher S. Fry, Sasidhar Uppuganti, Jeffry S. Nyman, John L. Fowlkes, Evangelia Kalaitzoglou

**Affiliations:** ^1^ Department of Pediatrics and Barnstable Brown Diabetes Center, University of Kentucky, Lexington, KY, United States; ^2^ Department of Biostatistics, College of Public Health, University of Kentucky, Lexington, KY, United States; ^3^ Center for Muscle Biology, University of Kentucky, Lexington, KY, United States; ^4^ Department of Orthopaedic Surgery, Vandebilt University Medical Center, Nashville, TN, United States; ^5^ Department of Orthopaedic Surgery, Vanderbilt University Medical Center and Department of Veterans Affairs, Tennessee Valley Healthcare System, Nashville, TN, United States

**Keywords:** type 1 diabetes, skeletal muscle, bone, myostatin, insulin

## Abstract

**Introduction:**

Type 1 diabetes is associated with deficits in both skeletal muscle and bone. Inhibition of myostatin, a negative regulator of muscle mass, was explored as a druggable target to improve the musculoskeletal phenotype associated with insulin-deficient diabetes in female mice.

**Methods:**

We investigated whether administration of an inhibitory myostatin antibody (MyoAb) in streptozotocin-induced diabetes in female mice is protective for skeletal muscle and bone. DBA/2J female mice were injected with low-dose streptozotocin or with citrate buffer (vehicle). Subsequently, mice were implanted with insulin-containing or vehicle pellets, with groups being randomized to myostatin or control antibody for 8 weeks. At study end, body composition and *in vivo* contractile muscle function were assessed, systemic myostatin and glycated hemoglobin were quantified, gastrocnemii were weighed and analyzed for fiber type composition, and femur microarchitecture and biomechanical properties were analyzed.

**Results:**

Glycated hemoglobin was significantly higher in diabetic mice compared to non-diabetic mice and diabetic mice treated with insulin. In diabetic mice, the combination of insulin and MyoAb resulted in higher lean mass, higher average gastrocnemius weight and larger muscle fiber size (Type IIB, IIX and hybrid fibers) compared to no treatment. *In vivo* contractile muscle function testing showed that insulin increased muscle torque in diabetic mice, however there was no effect of the MyoAb. Lastly, microarchitecture analysis of the distal femur showed improvement in some, but not all trabecular bone properties, in mice treated with insulin alone or together with MyoAb. Specifically, trabecular thickness and trabecular bone volume fraction were higher with combination treatment compared to insulin treatment alone.

**Conclusions:**

Myostatin inhibition when used in conjunction with insulin treatment improves muscle mass and trabecular bone properties in a mouse model of insulin-deficient diabetes in female mice.

## Introduction

Insulin-deficient diabetes or type 1 diabetes (T1D) is associated with deficits in skeletal strength and muscle mass (1–3) as well as increased risk for fracture (4–6). These complications involving the musculoskeletal system are likely a result of multiple factors that affect skeletal muscle and bone, including low insulin and IGF-1 levels, hyperglycemia and advanced glycation end-products (AGEs), diagnosis of T1D in childhood or adolescence resulting in inability to attain optimal muscle and bone mass during early adulthood and duration of diabetes, amongst others (3).

Secreted molecules from skeletal muscle, termed myokines, have been assessed in those with T1D and are found in different levels compared to healthy individuals (7–10). Myostatin, a myokine that is a negative regulator of muscle mass (11) and bone mass (12), has been shown to be elevated in the serum of those with T1D compared to healthy controls (7, 8). Due to its potent role in skeletal muscle development and its additional direct action on bone, inhibiting myostatin in T1D might help ameliorate the negative effects of diabetes on both muscle and bone.

Indeed, our previous studies have shown that pharmacologic inhibition of myostatin with an inhibitory myostatin antibody (MyoAb) is associated with higher body weight and lean mass, and better bone material properties and bone morphology in male insulin-deficient, diabetic mice when compared to treatment with a control antibody (13). The rate and severity of T1D complications exhibit sexual dimorphism, with some studies reporting that females experience a higher risk of cardiovascular complications (14) and worse metabolic control (15), whereas males have a higher risk of diabetic nephropathy (16). Due to concerns around sex-dependent outcomes in diabetes, we sought to evaluate whether the inhibition of myostatin in combination with insulin therapy would result in similar effects on the skeletal muscle and bone phenotype of female, insulin-deficient diabetic mice, compared to what we have previously shown in male mice.

## Methods

### Mouse study design

*Induction of diabetes and treatment arms:* Nine-week old female DBA/2J mice (n=8-10/group, The Jackson Laboratory, BarHarbor, ME) were injected with streptozotocin (Sigma Aldrich, Burlington, MA, USA) at 40 mg/kg/day in citrate buffer (diabetic-D) or with citrate buffer alone (non-diabetic-ND) intraperitoneally for five consecutive days, as previously described (13). Attrition rates varied across groups at study end (see limitations section). After confirming persistent hyperglycemia (non-fasting blood glucose above 250 mg/dl), diabetic (D) mice were randomized to receive sustained release LinBit insulin implants (Ins) (LinShin, Canada, Inc) or blank palmitic acid micro-crystal implants as control (Pal) (LinShin, Canada, Inc) while non-diabetic (ND) mice received palmitic acid containing LinBit implants (Pal) under anesthesia, as reported in our previously published methods (13). All implants were inserted and replaced based on manufacturer’s recommendations (http://www.linshincanada.com/linbit.html). Diabetic (D) and non-diabetic (ND) mice were further randomized to receive anti-myostatin (REGN647-MyoAb, Regeneron, Tarrytown, NY, USA) or Isotype control (REGN1945-ConAb, Regeneron) antibody at 10 mg/kg twice/week for 8 weeks, which were given subcutaneously after brief anesthesia with isoflurane, as previously described (13). The REGN647-MyoAb is highly specific to myostatin and effectively inhibits myostatin at the recommended dose in previous studies that have evaluated skeletal muscle and bone (17–19). Mouse weight was measured weekly and prior to euthanasia. For euthanasia, we use the open drop method of isoflurane anesthesia using 20% isoflurane in propylene glycol. After deep anesthesia induction as judged by non-responsiveness to a painful stimulus (tail pinch), mice are decapitated using surgical scissors. Gastrocnemius weight was measured after euthanasia. All mice were maintained in a 14-hour light:10-hour dark cycle and provided *ad libitum* access to chow diet (2018 Teklad, Envigo, Indianapolis, USA) and water throughout the study. All animal procedures were approved by the University of Kentucky Institutional Animal Care and Use Committee.

### Serum assays

During euthanasia whole blood was collected and stored at -20°C or processed for serum isolation. Serum specimens were stored at -20°C until ready to be assayed. Myostatin was measured in serum with a GDF-8/Myostatin Quantikine ELISA kit (Cat #: DGDF80, R&D systems/Biotechne, NE Minneapolis, MN, USA), Procollagen type 1 N-terminal propeptide (P1NP) was measured with Rat/mouse P1NP EIA assay kit (Euroimmun, Mountain Lakes, NJ, Cat # AC-33F1) and Cross Linked C-telopeptide of Type 1 Collagen (CTX-1) was measured with a mouse CTX-1 ELISA kit (ThermoFisher, Waltham, MA, Cat # EEL219). Glycated hemoglobin was measured in whole blood with an enzymatic mouse Hemoglobin A1c assay kit (Crystal Chem, Elk Grove Village, IL, USA, Cat # 80310).

### Body composition analysis

Animal body composition was evaluated by Echo-MRI™ (EchoMRI-100 (EMR-102 2016)) scans and parameters including total body fat, lean mass, and total body water were reported at the beginning of the study and prior to euthanasia, as previously described (13). During the scans, conscious mice were individually restrained in a clear cylindrical plastic holder (sized by animal weight). Each scan lasted approximately 2 minutes.

### Immunohistochemistry/fiber type and size analysis

The right gastrocnemii were excised, covered with O.C.T. Compound and mounted at resting length. They were frozen in liquid nitrogen-cooled isopentane and stored at −80°C until cryosectioning. Using a cryostat (HM525-NX, Thermo Fisher Scientific, Waltham, MA, USA), 7 μm-thick sections were cut and air dried for 1 h. Sections were stored at −20°C before IHC staining. Subsequently, for immunofluorescent assessment of muscle fiber type distribution and fiber type-specific cross-sectional area (CSA), unfixed cryosections were incubated overnight at 4°C in primary antibodies against myosin heavy chain (MyHC) type 1 (dilution 1:100, Developmental Studies Hybridoma Bank (DHSB), Cat#BA-D5 IgG2b), 2A (dilution 1:100, DSHB, Cat# SC-71 IgG1) and 2B (dilution 1:100, DSHB, Cat#BF-F3 IgM) in addition to laminin to visualize fiber borders (rabbit IgG, dilution 1:200; Millipore Sigma, Cat # L9393). MyHC type 2X expression was inferred from unstained fibers. On the following day, slides were washed in PBS and incubated for 90 minutes at room temperature in fluorescent-conjugated secondary antibodies (goat anti-mouse IgG2b, Alexa Fluor 647 secondary antibody (1:250; Invitrogen, Cat# A21242), goat anti-mouse IgG1, Alexa Fluor 488 secondary antibody (1:500; Invitrogen, Cat# A21121), goat anti-mouse IgM, Alexa Fluor 555 secondary antibody (1:250; Invitrogen, Cat# A21426) and goat anti-rabbit IgG, AMCA conjugated secondary antibody (1:150; Vector Laboratories, Cat#Cl-1000)) in PBS. Sections were post-fixed in methanol prior to mounting. Images were captured at 10x with an upright microscope (AxioImager M1; Zeiss, Göttingen, Germany). MyoVision software was used for automated analysis of fiber type distribution, and fiber type-specific cross-sectional area calculations (20).

### *In vivo* plantar flexor peak torque measurement

Prior to euthanasia, muscle function was assessed in a subgroup (n=4-5/group) of diabetic (D) mice. The strength of the plantar flexor muscle complex was assessed by *in vivo* isometric peak tetanic torque, similar to our prior published methods (13, 21). Briefly, in an induction chamber, mice were anesthetized with 2.5% isoflurane vaporized in 1.5 L/min oxygen (VetEquip vaporizer). Mice were then transferred to a secure nose cone with a continuous flow of isoflurane in oxygen. The right hind limb was analyzed for all mice, and fur was trimmed (Wahl Bravmini, Wahl Corporation) to ensure unobstructed electrode placement. Mice were placed in the supine position on a 37°C temperature regulated platform (809c *in-situ* mouse apparatus, Aurora Scientific, Aurora, ON, Canada), and the hind limb was secured using a clamp at the knee with the foot placed in a footplate on a dual-mode lever and motor (300D-300C-LRFP, Aurora Scientific). Surgical tape was wrapped around the foot secured to the footplate to prevent movement of the heel of placement shifting, and the footplate and motor arm was adjusted to place the tibia parallel with the platform with a 90-degree angle at the ankle. Needle electrodes were positioned percutaneously slightly lateral to the knee to maximally stimulate the tibial nerve using an electrical stimulator (High Power Bi-Phase Stimulator, Aurora Scientific). Using repeated twitches with the Instant Stimulation function with Live View in Dynamic Muscle Control LabBook (DMC v6.000), placement of needle electrodes was adjusted to optimize location to generate maximum isometric torque and eliminate antagonistic dorsiflexion. Once probe placement occurred, a series of progressive twitches were performed to determine optimal amperage to be used for the force-frequency experiment, with the goal of determining the lowest amperage to achieve the maximal twitch force output. Optimal amperage to produce maximal torque was determined by a progressive series of twitch experiments (0.05 s stimulus duration) beginning with 10 mA and increasing in small increments until the maximum torque stimulated by the minimum amperage was recorded with a maximum number of attempts set at 5. The amperage then remained constant throughout the force-frequency experiment (10, 40, 80, 120, 150, 180, and 200 Hz, 0.25s stimulus duration with a 2-minute rest period between each stimulus) from which isometric peak tetanic torque was recorded. Peak torque data were collected using DMC v6.000 and analyzed with Dynamic Muscle Analysis software (DMA v5.501). Plantar flexor isometric tetanic torque is reported with a force- frequency curve (with and without adjustment for mouse body weight).

### Micro-computed tomography analysis

Following euthanasia, the left femurs were stored in phosphate buffered saline (PBS) at −80°C. Following previously published methods (22, 23), the mid-point of the femur diaphysis and the distal femur metaphysis were scanned in PBS at room temperature using *ex vivo* μCT scanner (Scanco μCT50, Scanco Medical AG, Brϋttisellen, Switzerland) and then evaluated to assess cortical structure (e.g., cortical thickness, Ct.Th, cross-sectional bone area, Ct.Ar, cross-sectional moment of inertia, I_min_), trabecular architecture (e.g. bone volume fraction, BV/TV, trabecular thickness, Tb.Th, trabecular number, Tb.N., connectivity density, Conn.D), and tissue mineral density of cortical and trabecular bone (Ct.TMD and Tb.TMD). For both scans (1.86 mm across the femur mid-point and 3.72 mm above the physis), the scanner settings were as follows: an isotropic voxel size of 6 μm, peak x-ray voltage of 70 kVp, tube current of 114 μA, integration time of 300 ms, sampling rate of 1160 acquisitions per 1000 projections per rotation of the tube holder. A 0.1 mm thick, aluminum filter was between the X-ray beam and bone to narrow the energy spectrum and minimize beam hardening effects. Furthermore, a manufacturer recommended beam hardening correction (as part of the calibration to the hydroxyapatite phantom) was applied during each scan. Specifically, a manufacturer provided quality control (QC) phantom (part no. A09200 Ø34 x 60 mm) with five packed columns of HA (Mean of rod 1, 2, 3, 4 and 5 being -15, 100, 210, 415, and 790 mg HA/cm^3^) was scanned on a weekly and monthly basis to ascertain that the x-ray attenuation was within ±5% of factory standard. All the scans were performed with this calibration file for a chosen x-ray energy setting and the manufacturer recommended beam hardening (BH) correction of 1200 mg HA/cm^3^.

Post-reconstruction of the scans by Scanco software, we applied a noise filter to the image stack (Gaussian smoothing parameters: standard deviation of the distribution, Sigma, and weighting of neighboring pixels, Support) of the diaphysis (Sigma = 0.8 and Support = 2) and metaphysis (Sigma = 0.2 and Support = 1). Then, segmentation of bone from soft tissue and air used different global density threshold for cortical bone (≥900.5 mgHA/cm^3^) and trabecular bone (≥429.4 mgHA/cm^3^) so that bone morphology and density parameters could be determined by Standard Scanco evaluation scripts.

### Three-point bend testing

Following the μCT evaluation of the femur mid-diaphysis, each hydrated femur was loaded-to-failure at 3 mm/min in three-point bending with a span of 8 mm using a mechanical testing system (DynaMight 8800, Instron, Norwood, MA). During the mechanical test of each bone, the anterior side faced down and the medial side forward. The resulting force (Honeywell load cell, P/N 060-0863-02, maximum capacity of 100N) vs. displacement (linear variable differential transducer of the linear actuator) data were acquired at 50 Hz and processed using a custom Matlab (Mathworks, Nack, MA) script to determine the stiffness, yield force, ultimate force, post-yield displacement (PYD), and work-to-failure (area under the force vs. displacement curve). The yield point was identified at the intersection of the force vs. displacement curve and a linear curve with a slope of 0.9 x stiffness originating from the origin. Using equations from beam theory and μCT structural parameters, we estimated modulus and ultimate stress. Toughness was 3 x work-to-fracture/Ct.Ar/Span (24).

### Statistical analysis

We summarize the mouse data using means and the standard deviation for continuous variables. In understanding the differences between the six groups, we compare the outcomes using the averages and represent these using graphs. We used the One-way Analysis of Variance (ANOVA) for comparing multiple groups for each outcome variable, followed by multiple comparisons with the Tukey method across the six mouse groups. The general linear model technique compares the six groups while evaluating the magnitude and the direction of each treatment for outcome variables.

We evaluated the normality of the continuous variables using both graphical and statistical methods. Specifically, we used quantile-quantile (Q-Q plots) to visually compare the distribution of the variables against a normal distribution. Given the small sample size within the groups, we applied the Shapiro-Wilk test to assess normality. To validate the results from the ANOVA, we conducted a Wilcoxon test, a non-parametric alternative. This non-parametric test was chosen due to its ability to handle non-normally distributed data, making no assumptions about the underlying distributions of the mice groups. A boxplot of the variables did not reveal any noticeable outliers. To further investigate the impact of potential outliers, we performed a sensitivity analysis. In cases where outliers were detected, we employed robust statistical methods, such as non-parametric tests, to reduce their influence on the results.

We conduct all statistical hypothesis tests at the standard 5% significance level with a rejection of the null hypothesis for p-values >0.05. The SAS version 9.4 (TS1M1 SAS Institute Inc., Cary, NC, USA) statistical software and Graph Pad Prism 10.4.0 version are used for all analyses.

## Results

### Insulin therapy resulted in lower glycated hemoglobin and higher bone formation marker P1NP, whereas MyoAb therapy resulted in lower systemic myostatin

**Figure 1:**
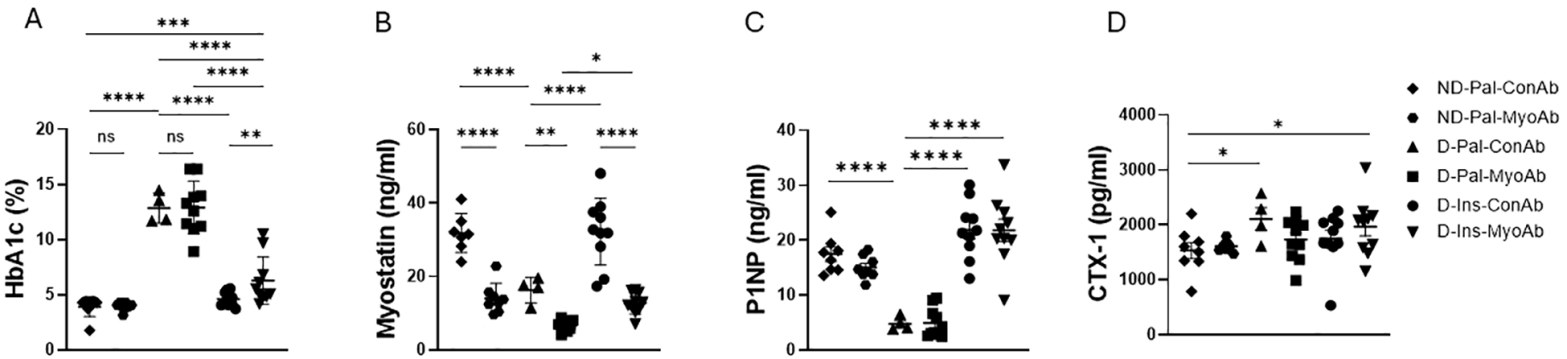
Effects of streptozotocin (STZ)-induced diabetes, insulin and MyoAb treatment on whole blood glycated hemoglobin (HbA1c) **(A)**, serum myostatin **(B)**, serum procollagen type 1 N-terminal propeptide (P1NP) **(C)** and plasma Cross Linked C-telopeptide of Type 1 Collagen (CTX-1) **(D)** at study end. Data presented as individual points with mean ± SD. *p<0.05, **p<0.01, ***p<0.001, ****p<0.0001, ns: not significant.

Glycated hemoglobin (HbA1c) at study end was significantly higher in mice with diabetes treated with vehicle (D-Pal) compared to non-diabetic mice (ND) and diabetic mice treated with insulin (D-Ins) (**Figure 1A**). Furthermore, HbA1c was higher in mice treated with combination of insulin and myostatin antibody compared to those treated with insulin alone (D-Ins-MyoAb vs D-Ins-ConAb, 6.3% vs 4.6%, p=0.008) (**Figure 1A**). Systemic myostatin was lower in D-Pal-ConAb (16.3 ng/ml) compared to ND-Pal-ConAb (31.8 ng/ml, p<0.001) or D-Ins-ConAb mice (15.9 ng/ml, p<0.001) (**Figure 1B**). As anticipated, all mice treated with MyoAb had lower detected serum myostatin levels, although the MyoAb could potentially interfere with the myostatin assay making the reductions in myostatin seen in the MyoAb treated mice partially artifactual. Bone formation marker P1NP was lower in diabetic mice compared to non-diabetic mice, while insulin-treated, diabetic mice appeared to have higher P1NP levels compared to diabetic mice not treated with insulin (D-Ins-ConAb vs D-Pal-ConAb, 21.9 ng/ml vs 4.8 ng/ml, p<0.0001) (**Figure 1C**). Bone resorption marker CTX-1 was higher in diabetic mice compared to non-diabetic mice, however insulin treatment was only associated with a trend for lower CTX-1 levels (D-Ins-ConAb vs D-Pal-ConAb, 1747.3 pg/ml vs 2109.8 pg/ml, p>0.1) (**Figure 1D**). There was no significant effect of the myostatin inhibitory antibody on P1NP or CTX-1 levels.

### Treatment with combination of insulin/MyoAb resulted in higher body weight and skeletal muscle mass compared to no treatment

**Figure 2:**
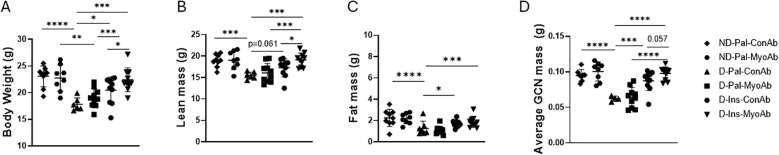
Effects of STZ-induced diabetes, insulin and MyoAb treatment on body weight **(A)**, lean mass **(B)**, fat mass **(C)** as measured by Echo MRI and average gastrocnemius mass **(D)** at study end. Data presented as individual points with mean ± SD. *p<0.05, **p<0.01, ***p<0.001, ****p<0.0001.

Diabetic mice had lower body weight, lean mass and fat mass compared to non-diabetic mice at study end (**Figures 2A–C**). Insulin treated diabetic mice had higher body weight at study end compared to non-insulin treated diabetic mice (D-Ins-ConAb vs D-Pal-ConAb, 20.5 g vs 17.8 g, p=0.019) (**Figure 2A**). Their lean mass was also higher than non-insulin treated diabetic mice at study end (D-Ins-ConAb vs D-Pal-ConAb,17. 3 g vs 15.5 g, p= 0.061) (**Figure 2B**). Diabetic mice treated with combination of insulin/MyoAb had higher body weight compared to diabetic mice treated with MyoAb or insulin alone (D-Ins-MyoAb vs D-Pal-MyoAb, 22.4 g vs 19 g, p<0.0001 and D-Ins-MyoAb vs D-Ins-ConAb, 22.4 g vs 20.5 g, p=0.04) (**Figure 2A**). Their lean mass was also significantly higher compared to mice treated with MyoAb or insulin alone (D-Ins-MyoAb vs D-Pal-MyoAb, 19.1 g vs 16.1 g, p<0.001 and D-Ins-MyoAb vs D-Ins-ConAb, 19.1 g vs 17.3 g, p=0.03) (**Figure 2B**). Lastly, fat mass was higher in non-diabetic mice compared to mice with diabetes, not treated with insulin, but similar to diabetic mice treated with insulin. No significant differences in fat mass were observed between MyoAb and ConAb groups (**Figure 2C**). Similarly to lean mass improving with combination therapy with insulin/MyoAb, average gastrocnemius weight showed greater improvements with combination treatment compared to MyoAb or insulin alone (D-Ins-MyoAb vs D-Pal-MyoAb, 0.1 g vs 0.06 g, p<0.001 and D-Ins-MyoAb vs D-Ins-ConAb, 0.1 g vs 0.09 g, p=0.056) (**Figure 2D**).

### Insulin +/- MyoAb treatment was associated with improved muscle fiber size in diabetic mice, whereas skeletal muscle strength partially improved with insulin therapy

**Figure 3:**
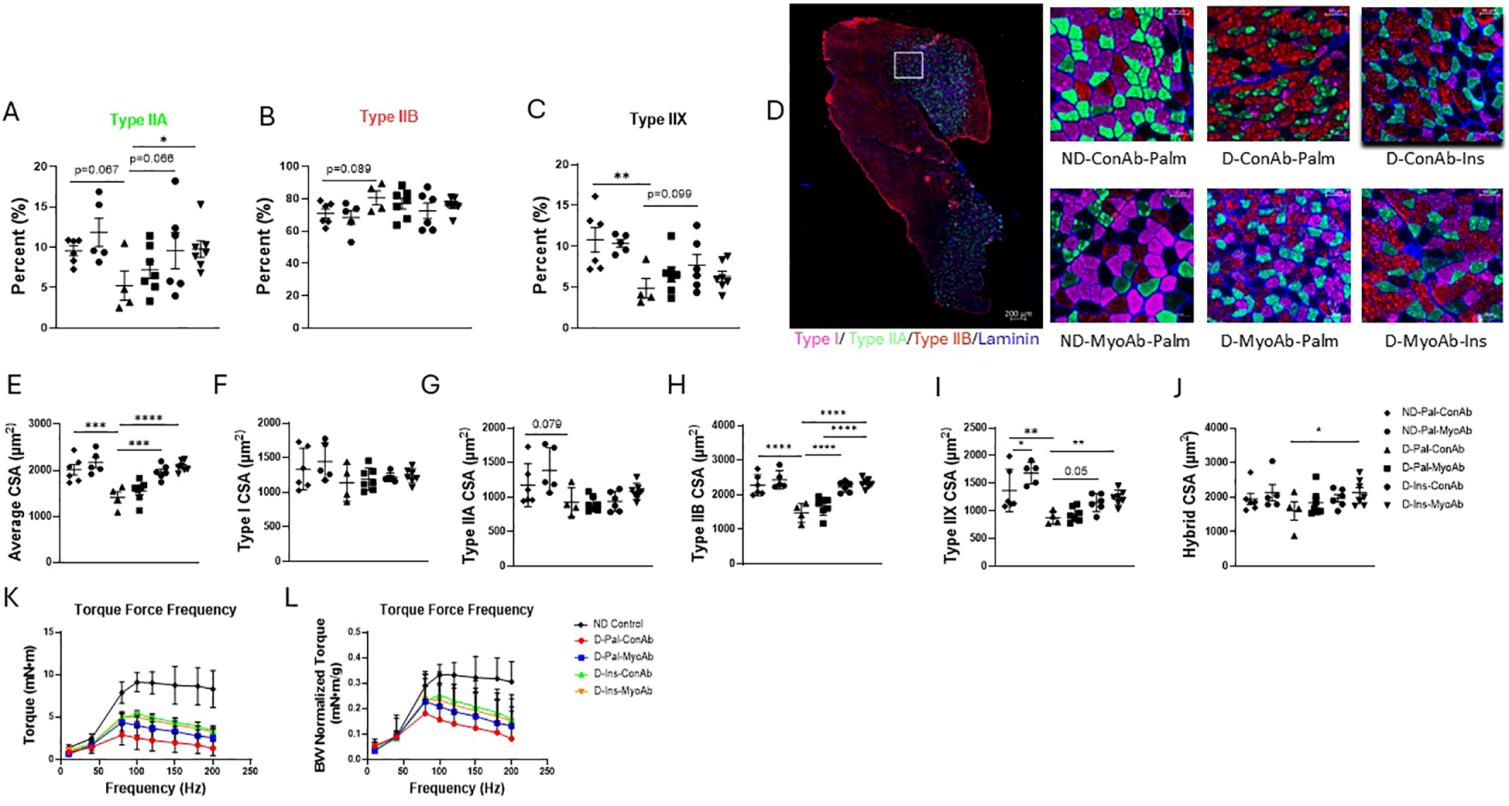
Effects of STZ-induced diabetes, insulin and MyoAb treatment on fiber percent **(A–C)** and cross-sectional area (CSA) **(E–J)** in gastrocnemius muscle. Representative images from immunohistochemical analysis of gastrocnemius muscle cross sections for myosin heavy chain (MHC) type I (pink), type IIA (green) and type IIB (red) **(D)**. Unstained fibers are MHC type IIX. Scale bar = 50 μm. Effects of STZ-induced diabetes, insulin and MyoAb treatment on torque-frequency curve **(K)** and torque-frequency curve adjusted for body weight **(L)** at study end. For K and L non-diabetic control mice were from a separate cohort that did not receive MyoAb or ConAb. Data presented as individual points with mean +/- SD. *p<0.05, **p<0.01, ***p<0.001, ****p<0.0001.

Fiber type staining did not show any differences in type I or hybrid fiber percentage because of diabetes status or any of the treatments (data not shown). Diabetic mice had a trend towards lower percentage of type IIA and higher percentage of type IIB fibers and significantly lower percentage of type IIX fibers compared to non-diabetic mice (ND-Pal-ConAb vs D-Pal-ConAb) (**Figures 3A–D**). Insulin treatment resulted in a trend for higher type IIA and IIX fiber percentage (**Figures 3A–D**). Combined treatment with insulin and MyoAb resulted in higher percentage of type IIA fibers in diabetic mice compared to vehicle (D-Ins-MyoAb vs D-Pal-ConAb, p=0.048, **Figures 3A, D**).

When evaluating the average fiber cross-sectional area (CSA) with no regard to specific fiber type, diabetic mice had significantly lower CSA compared to non-diabetic mice, whereas diabetic mice on insulin had similar fiber CSA to non-diabetic mice (**Figure 3E**). The MyoAb did not appear to significantly increase average CSA (**Figure 3E**). No changes were observed in the cross-sectional area (CSA) of type I fibers as result of diabetes or any of the treatments (**Figures 3F, D**). Diabetic mice had a trend for smaller CSA of type IIA fibers (**Figures 3G, D**) and significantly smaller CSA in type IIB and IIX fibers compared to non-diabetic mice (**Figures 3H, I, D**). Insulin treatment was associated with larger type IIB and IIX (**Figures 3H, I, D**) but not type IIA fiber CSA compared to no insulin treatment (**Figures 3D, G**). MyoAb and insulin combined treatment was associated with larger CSA of hybrid fibers when compared to mice treated with neither (D-Ins-MyoAb vs D-Pal-ConAb, 2134 µm^2^ vs 1605 µm^2^, p=0.046) (**Figures 3D, J**).

*In vivo* contractile muscle function testing showed that insulin treatment compared to vehicle was associated with increased raw muscle torque in diabetic mice (D-Ins-ConAb vs D-Pal-ConAb, p=0.04, **Figure 3K**), however, this effect was no longer significant when correcting muscle torque to body weight (**Figure 3L**). Therefore, this suggests that insulin improved muscle torque due to an increase in lean mass/body weight. Lastly, there was no further benefit in muscle strength with the addition of MyoAb to insulin treatment (D-Ins-ConAb vs D-Ins-MyoAb, **Figure 3K**).

### Several cortical and trabecular bone parameters and ultimate force were superior in insulin +/- MyoAb treated diabetic mice compared to no treatment

**Figure 4:**
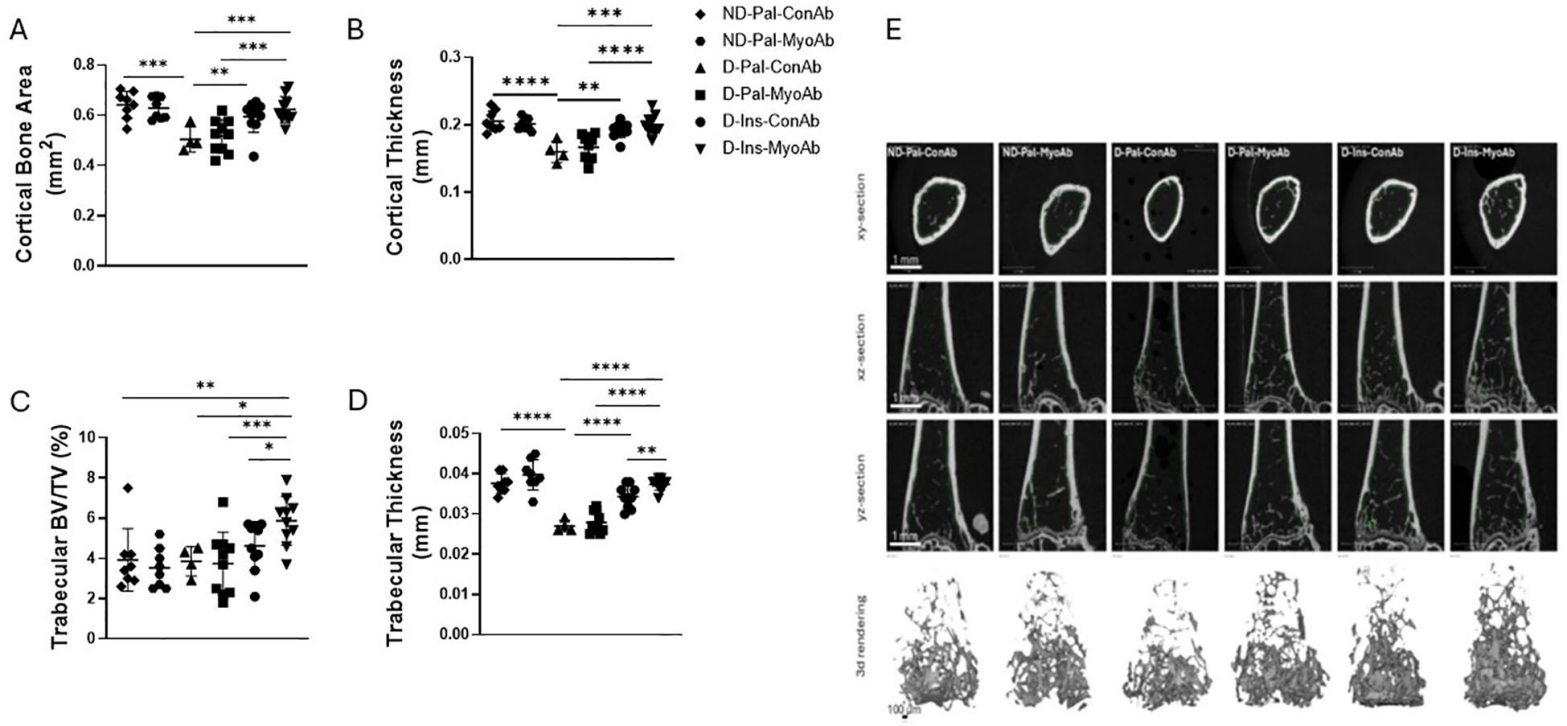
Effects of STZ-induced diabetes, insulin and MyoAb treatment on bone microarchitecture. Selected cortical properties, including Cortical Bone Area **(A)** and Cortical Thickness **(B)**; selected trabecular properties including Bone Volume Fraction **(C)** and Trabecular Thickness **(D)** as measured by microCT analysis. Representative images of cortical and trabecular bone microarchitecture by microCT **(E)**. Data presented as individual values with mean ± SD. *p<0.05, **p<0.01, ***p<0.001, ****p<0.0001.

µCT analysis of the femur showed that cortical properties, such as cortical bone area, cortical thickness and cortical porosity, were superior in mice treated with insulin treatment compared to non-insulin treated mice, however the addition of MyoAb did not further improve these parameters (**Figures 4A, B**, **Table 1**). In the trabecular compartment there was improvement in some, but not all bone properties, in mice treated with insulin alone or combined with MyoAb (**Figure 4**, **Table 1**). Specifically, trabecular bone volume fraction was improved with combination treatment of insulin/MyoAb compared to insulin treatment alone (D-Ins-MyoAb vs D-Ins-ConAb, 5.87% vs 4.61%, p=0.03) (**Figure 4C**, **Table 1**), as was trabecular thickness (D-Ins-MyoAb vs D-Ins-ConAb, 40 µm vs 30µm, p=0.009) (**Figure 4D**, **Table 1**).

**Table 1:** MicroCT trabecular and cortical parameters of femur, including whole bone mechanical properties of femur from 3-point bending test.

Bone Parameter	ND-Palm-ConAb	ND-Palm-MyoAb	D-Palm-ConAb	D-Palm-MyoAb	D-Ins-ConAb	D-Ins-MyoAb	Diabetes effect (1 vs 3)	Insulin effect (3 vs 5)	MyoAb effect (1 vs 2 or 3 vs 4)	Insulin/MyoAb effect (3 vs 6)	MyoAb additive effect (5 vs 6)
Diaphysis/Cortical bone											
Ma.V (mm^3^)	0.72 ±0.28	0.75 ±0.29	0.99 ±0.09	0.95 ±0.1	0.75 ±0.26	0.83 ±0.76	p=0.038	P=0.056	ns	ns	ns
Imin (mm^4^)	0.055 ± 0.01	0.053 ± 0.01	0.047 ± 0.01	0.045 ± 0.01	0.051 ± 0.01	0.053 ± 0.01	P=0.069	ns	ns	ns	ns
Ct. Ar (mm^2^)	0.64 ±0.05	0.63 ±0.04	0.50 ±0.05	0.52 ±0.07	0.59 ±0.06	0.62 ±0.05	P<0.001	P=0.009	ns	P<0.001	ns
Tt. Ar(mm^2^)	1.09 ±0.07	1.09 ±0.07	1.04 ±0.05	1.03 ±0.07	1.05 ±0.11	1.08 ±0.06	ns	ns	ns	ns	ns
Ct. Th (mm)	0.21 ±0.02	0.20 ±0.01	0.16 ±0.02	0.17 ±0.02	0.19 ±0.01	0.20 ±0.01	P<0.001	P<0.001	ns	P<0.001	ns
Ct. TMD (mgHA/cm^3^)	1325 ± 18	1309 ±5	1306 ±11	1313 ±13	1318 ±14	1303 ± 14	P=0.025	ns	P=0.017 (1 vs 2)	ns	P=0.018
Ct. Po (%)	4.05 ±0.41	4.08 ±0.13	4.79 ±0.43	4.67 ±0.61	4.08 ±0.13	4.19 ±0.28	P=0.003	P=0.003	ns	P=0.01	ns
Length (mm)	13.8 ±0.3	13.6 ±0.5	13.4 ±0.2	13.4 ±0.4	13.4 ±0.5	13.6 ±0.3	P=0.082	ns	ns	ns	ns
Ultimate stress (MPa)	240 ±35	273 ±13	257 ±52	252 ±38	278 ±27	265 ±34	ns	ns	0.049(1 vs 2)	ns	ns
Modulus (GPa)	16.28 ± 2.76	18.27 ± 1.50	19.51± 3.37	16.58 ± 2.97	17.6 ±1.85	18.18 ± 3.18	P=0.052	ns	P=0.067(3 vs 4)	ns	ns
Stiffness (N/mm)	83.09 ± 12.0	91.17 ± 11.0	85.15 ± 13.3	70.86 ± 14.8	86.64 ± 14.4	90.66 ± 15.3	ns	ns	P=0.09(3 vs 4)	ns	ns
Ultimate Force (N)	13.17 ± 1.53	14.89 ± 1.36	12.01 ± 2.27	11.70 ± 2.11	14.41 ± 1.41	14.36 ± 2.1	ns	P=0.03	P=0.063(1 vs 2)	P=0.033	ns
PYD (mm)	0.23 ±0.20	0.41 ± 0.18	0.25 ±0.39	0.11±0.14	0.20 ±0.16	0.27 ±0.17	ns	ns	P=0.069(1 vs 2)	ns	ns
Metaphysis/Trabecular Bone											
Tb. BV/TV (%)	3.88 ±1.56	3.53 ±0.98	3.85 ±0.73	3.72 ±1.54	4.61 ±1.2	5.87 ±1.19	ns	ns	ns	P=0.011	P=0.034
Conn.D (mm^-3^)	174.2 ± 63.0	127.7 ± 48.1	246.7 ± 72.9	262.2 ± 146.6	282.0 ± 118.8	316.3 ± 98.9	ns	ns	ns	ns	ns
SMI	2.98± 0.37	2.94± 0.28	2.55± 0.15	2.71± 0.36	2.790.24±	2.51± 0.23	P=0.021	ns	ns	ns	P=0.039
Tb.N(mm^-1^)	2.68± 0.33	2.54± 0.15	2.98± 0.21	2.88± 0.36	2.95± 0.41	3.01± 0.39	ns	ns	ns	ns	ns
Tb.Th (mm)	0.038± 0.002	0.040±0.004	0.027±0.001	0.028±0.002	0.034±0.003	0.037±0.002	P<0.001	P<0.001	ns	<0.001	P=0.009
Tb.Sp (mm)	0.39± 0.05	0.41± 0.02	0.34± 0.02	0.36 ± 0.04	0.36± 0.06	0.35± 0.04	P=0.09	ns	ns	ns	ns
Tb. TMD (mgHA/cm^3^)	782.2± 29.2	796.9± 20.3	712.4± 15.6	725.0± 28.0	770.7± 32.5	788.4± 20.4	P<0.001	P<0.001	ns	<0.001	ns

Data is presented as mean ± SD. Abbreviations: BV/TV, bone volume/tissue volume; Conn.D, connectivity density; Ct.Ar, cortical area; Ct.Po, cortical porosity; Ct.TMD, cortical tissue mineral density; I_min_, minimal moment of inertia; Ma.V, marrow volume; PYD, post yield deflection; SMI, structural model index; Tb.N, trabecular number; Tb.Sp, trabecular separation; Tb.Th, trabecular thickness; Tb.TMD, trabecular tissue mineral density; Tt.Ar, total cross-sectional area; Ultimate Force: maximum value of load attained during the test, PYD, Post-yield displacement. p values as indicated for comparisons between indicated groups, ns, not significant.

Interestingly, three-point bending testing did not show an effect of diabetes in most measured parameters, apart from modulus (**Table 1**). Despite this, insulin treatment was associated with higher ultimate force compared to no insulin treatment (D-Palm-ConAb vs D-Ins-ConAb, **Table 1**). Furthermore, MyoAb was associated with effects in mechanical properties of the bone that seemed to be depending on the status of diabetes. In non-diabetic mice, there was a trend for higher ultimate stress (bending strength) and superior ultimate force (structural-dependent bending strength) and post-yield displacement as a result of treatment with MyoAb (ND-Pal-ConAb vs ND-Pal-MyoAb, **Table 1**), whereas in diabetic mice without insulin MyoAb did not affect the strength of the femurs (D-Pal-ConAb vs D-Pal-MyoAb, **Table 1**).

## Discussion

T1D is associated with increased fracture risk (5, 6, 25) and impaired skeletal muscle mass and function (2, 26). Skeletal muscle and bone communicate under healthy conditions (27, 28) but also during disease states, including diabetes (3). Myostatin is a secreted myokine with direct effects on bone (29). It acts as a negative regulator of skeletal muscle mass (11) and bone mass (30–32). Recently, serum levels of myostatin were found to be elevated in humans with T1D (7, 8), however, the significance of this elevation is still unclear. Given its role as a negative regulator of skeletal muscle and bone, its inhibition offers a targeted intervention that could be beneficial for diabetic bone and muscle (33–35).

Contrary to what has been observed in humans with T1D who have higher myostatin levels (7), in our study, female insulin-deficient, diabetic mice, did not have higher systemic myostatin levels compared to non-diabetic mice. This was true even after adjusting myostatin for lean mass. This finding is consistent with our findings in male, insulin-deficient mice where myostatin levels were lower in diabetic mice compared to non-diabetic mice (13). Additionally, we observed that insulin treatment is associated with higher myostatin levels, similar to the non-diabetic state. This is contrary to what has been observed in diabetic rats where no change in myostatin transcripts was observed with streptozotocin-induced diabetes or insulin treatment (36). Given contradictory findings, further studies are needed to fully elucidate whether insulin and the diabetic state have direct effects on myostatin expression and secretion.

In this study, insulin monotherapy of female, diabetic mice resulted in higher body mass, lean mass and bone formation marker P1NP, and improved bone microarchitecture and biomechanical properties, which is in accordance with previous studies in insulin-deficient female mice (37). Despite myostatin levels being lower in diabetic mice, our study shows that inhibiting myostatin with MyoAb has positive effects for skeletal muscle and bone when combined with insulin. Furthermore, in this study, MyoAb monotherapy was associated with small benefits in the biomechanical bone properties of non-diabetic mice, however, in diabetic mice some of the biomechanical properties are not affected or are negatively affected by the antibody. Lastly, treatment with MyoAb did not result in gains in muscle mass, muscle strength or improved bone trabecular properties in diabetic mice, unless combined with insulin. Insulin and MyoAb combined therapy resulted in better muscle torque, however this is likely due to higher lean mass and not due to improved muscle quality in the treated groups. Indeed, this lack of improvement in specific force has previously been reported by other groups that have used MyoAb therapy in mice (19).

Insulin therapy combined with MyoAb increased the ultimate force that the femur mid-diaphysis experienced during the load-to-failure test likely because combined therapy increased cortical thickness and decreased cortical porosity. Ultimate stress, which is an estimate of the material strength of cortical bone, likely did not improve because tissue mineral density of cortical bone was not affected with combination treatment. These results are in accordance with our previous studies showing positive effects of combination treatment of insulin and myostatin inhibition on the musculoskeletal phenotype of male diabetic mice (13). However, male diabetic mice showed improvements in skeletal muscle and bone parameters both with MyoAb monotherapy and combined insulin/MyoAb therapy (13). In that study, we reported that MyoAb resulted in changes in genes involved in the Wnt pathway in skeletal muscle from male diabetic mice (13). Additionally, we showed decreased Smad2 phosphorylation in osteoblasts treated with the MyoAb *in vitro* (13), supporting direct effects of the MyoAb on bone.

The changes in cortical bone properties observed with MyoAb monotherapy only in male mice could be related to the skeleton of female mice being more resistant to the negative effects of insulin deficiency and positive effects of MyoAb treatment on bone size and strength. In addition, female mice could require different antibody dosing for its effects to be apparent in this bone compartment. Indeed, myostatin has been shown to be reduced in male mice as a result of growth hormone regulation (38), therefore, its inhibition with MyoAb could be potentiated in male compared to female mice explaining the limited response to the MyoAb monotherapy in female mice. Additionally, human studies have shown increased mRNA of the activin receptor IIB (AcvRIIB) gene in women compared to men (39), which could account for increased myostatin activity in females and negative regulation of skeletal muscle size. Lastly, estradiol signaling in skeletal muscle has been implicated in myostatin regulation and myostatin has been shown to mostly affect carbohydrate metabolism pathways in male skeletal muscle, whereas in females it mostly affects oxidative metabolism pathways (40). These studies support a sex-specific susceptibility to myostatin, which likely explains the more robust response to MyoAb monotherapy in our previous study involving male diabetic mice.

Coleman et al. reported improvements in insulin sensitivity and glycemic control with myostatin inhibition in a type 1 diabetes (Akita) animal model in male mice (33). In our study, we did not notice significant changes in glycemic control between diabetic mice treated with MyoAb and the control antibody in the groups not receiving insulin, although some diabetic mice receiving the control antibody group were found deceased, likely due to uncontrolled hyperglycemia. However, the glycemic control between insulin treated groups was not identical, as the group receiving myostatin antibody (D-Ins-MyoAb) had higher glycated hemoglobin than the group receiving control antibody (D-Ins-ConAb). This finding contrasts with *in vivo* studies that have shown lower glucose levels in obese mice injected with a neutralizing antibody to myostatin (41) or protection from insulin resistance in mice with loss of function mutation of myostatin (42). In contrast, other studies have shown that inhibiting myostatin by administration of a soluble activin receptor type IIB, which is myostatin’s primary receptor, does not improve glycemic control of insulin-deficient mice (43). Furthermore, *in vitro* studies have shown that myostatin can promote glucose consumption and uptake and increase glycolysis in skeletal muscle cells through upregulating genes involved in glucose metabolism (44), supporting the theory that myostatin promotes glucose metabolism. Additionally, we cannot rule out an interaction between exogenous insulin and MyoAb that could interfere with insulin signaling, potentially blocking the AMP-activated protein kinase pathway that has been shown to be regulated by myostatin in skeletal muscle cells (44). Adding to the complexity of myostatin and insulin interplay is IGF-1, a growth factor with similar molecular structure to insulin, which has been shown to be decreased in T1D (45). IGF-1 has been shown to suppress myostatin signaling during myogenesis (46) and could offer an additional target for intervention in muscle-bone cross talk in T1D. Future mechanistic studies are needed to clarify the metabolic function of myostatin and its interaction with insulin and related growth factors, such as IGF-1.

Several studies have evaluated various treatments for diabetes-induced muscle atrophy, with some specifically targeting the myostatin pathway in skeletal muscle and the Wnt pathway in bone. Among those, low-intensity pulsed ultrasound for diabetes associated muscle atrophy in rats was beneficial as it downregulated myostatin and AcvRIIB expression in skeletal muscle (47). C-peptide has also been shown to protect against skeletal muscle atrophy in insulin-deficient, diabetic rats with effects on Atrogin 1 and Traf6 expression (48), as has alpha- lipoic acid (49). Additionally, physical exercise has been shown to be beneficial for skeletal muscle health in insulin-deficient diabetic animals (50) as well as in humans (51, 52). Similar benefits have been seen with exercise in the bone phenotype of insulin-deficient rodents (35) and humans with type 1 diabetes (53, 54). Several anabolic treatments have been studied for their potential role against diabetic bone disease. These have included IGF-1, which resulted in improvements in weight gain and growth in diabetic rats, when used alone or in combination with insulin (55), sclerostin inhibition which lead to improvements in bone healing in a fracture model of insulin-deficient diabetes in mice by altering the Wnt pathway (56) and teriparatide and abaloparatide which increased bone mass and improved bone strength and bone turnover in mice with STZ-induced diabetes (57). Most of these interventions have reported on either skeletal muscle or bone in the context of type 1 diabetes, therefore our approach is unique as it was designed to evaluate skeletal muscle and bone simultaneously. More importantly, most of the existing literature regarding interventions to improve skeletal muscle and bone in type 1 diabetes is based on insulin-deficient male mice, whereas our study includes female mice.

Our study has several limitations. Although we started this study with 8–10 mice/group, several mice from the untreated group with diabetes on the control antibody (D-Pal-ConAb) did not complete the study, which resulted in missing or partial data. Specifically, among the ND-Pal-ConAb mice, the attrition rate was 1/10, while the attrition rate for the ND-Pal-MyoAb control group was 1/9. The highest attrition rate occurred in the D-Pal-ConAb group, with an attrition rate of 3/9. The cause of death for these mice remains unclear, and as a result, data from these mice were excluded from the analysis. However, we compared the baseline characteristics of the mice that were included in the study with those that were excluded to assess the impact of attrition. Our review revealed that the baseline characteristics of the mice that dropped out were similar to those that remained in the study until its conclusion. Therefore, we believe that the dropouts were not attributed to systematic bias, but rather to factors such as uncontrolled diabetes. These mice were either euthanized due to having distended abdomen or were found deceased during the study. We speculate that this was likely due to poorly controlled diabetes as they were not receiving insulin therapy. Furthermore, two mice from the D-Pal-ConAb group were found dead during the final week of the study resulting in partial data collection and one mouse from the D-Pal-ConAb group was excluded from analysis due to exhibiting very mild hyperglycemia (incomplete diabetes). Interestingly, the attrition of mice that were not treated with insulin but received the myostatin blocking antibody (D-Pal-MyoAb) was not affected.

Another limitation to our study is that the glycemic control between insulin treated groups was not identical, as the group receiving myostatin antibody had higher glycated hemoglobin than the group receiving the control antibody. This difference in glycemic control between the myostatin and control antibody-treated mice was not observed in the other groups (non-diabetic and diabetic without insulin) and also not observed in our previous study when male mice were treated with this antibody (13), therefore we cannot exclude that there could have been suboptimal glycemic control with insulin pellets in some of the mice with higher HbA1c. Lastly, we performed muscle function testing on a cohort of non-diabetic female mice that were not treated with the MyoAb or ConAb to use as a reference of normal muscle function.

## Conclusions

Inhibition of myostatin with an antibody combined with insulin therapy, appears to have beneficial effects to the skeletal muscle and trabecular bone of female, insulin-deficient diabetic mice. Combined therapy also improved cortical thickness while reducing cortical porosity, and therefore, increased structural-dependent bending strength of the femur mid-diaphysis. It did not, however, affect tissue mineral density and the estimated material strength of cortical bone. Future studies should evaluate the mechanism by which myostatin interacts with insulin, with specific focus on how this interaction is affected by sex-specific factors.

## Data Availability

The raw data supporting the conclusions of this article will be made available by the authors, without undue reservation.
